# miR-34a negatively regulates cell cycle factor Cdt2/DTL in HPV infected cervical cancer cells

**DOI:** 10.1186/s12885-022-09879-5

**Published:** 2022-07-15

**Authors:** Garima Singh, Sonika Kumari Sharma, Samarendra Kumar Singh

**Affiliations:** Cell Cycle and Cancer Laboratory, School of Biotechnology, Institute of Science, Banaras Hindu University, Varanasi, UP-221005 India

**Keywords:** Cervical cancer, High-risk HPV, Cdt2, miR-34a, HPV E6

## Abstract

MicroRNAs have emerged as an important regulator of cell cycle and various other cellular processes. Aberration in microRNAs has been linked with development of several cancers and other diseases but still very little is known about the mechanism by which they regulate these cellular events. High risk human papilloma virus (HR HPV) is the causative agent of 99% of cervical cancer cases which attenuates multiple tumor suppressors and checkpoint factors of the host cell. The viral proteins also stabilize many oncogenic factors, including an essential cell cycle regulator Cdt2/DTL which in turn promotes cell transformation and proliferation. In this study, we report that a micro-RNA, miR-34a by suppressing HPV E6 protein, destabilizes Cdt2/DTL protein level in HPV infected cervical cancer cell lines. Destabilization of Cdt2 stabilizes pro-apoptotic and onco-suppressor proteins like p21 and Set8 and suppresses cell proliferation, invasion and migration capabilities of the HPV positive cervical cancer cells. Overexpression of either HPV E6 or Cdt2 genes along with miR-34a restored back the suppressed proliferation rate. This study is the first-ever report to show that miR-34a regulates cell cycle factor Cdt2 by suppressing viral E6 protein level, thus opening up the possibility of exploring miR-34a as a specific therapy for cervical cancer treatment.

**Supplementary Information:**

The online version contains supplementary material available at 10.1186/s12885-022-09879-5.

## Background

With 6,04,000 new cases and 3,42,000 deaths annually, cervical cancer stands as the fourth most common cause of cancer death among women worldwide [1]. Among all the cervical cancer cases, 99% of them are attributed to high risk human papillomavirus (HPV) infection [2] and most of these cases are prevalent in developing countries like India. Due to lack of awareness in these countries, use of vaccines is negligible (no data available) and therefore alternate therapeutic options could be a significant solution. Apart from cervical cancer, HPV is also known to cause penile, vulvar, anal, vaginal, oropharyngeal and head and neck cancers [3].

HPV is a non-enveloped double-stranded DNA virus which eventually integrates into the host genome upon infection and causes oncogenic transformation and hyperproliferation of the cells [4]. Out of 15 HPVs categorized as high risk (HR) till date, HPV 16 and HPV 18 are responsible for almost 70% of all the cervical cancer cases [2]. The HR HPV encoded oncoproteins E5, E6 and E7 are the primary factors responsible for oncogenic transformation and progression of cervical cancer. The E7 protein interacts with retinoblastoma family proteins (RB1, RBL1 and RBL2) and degrades them to release E2F [5, 6] whereas E6 proteins causes ubiquitin-mediated degradation of p53 via E6AP, ubiquitin ligase [7]. Apart from regulating multiple genes and proteins to inhibits apoptotic signaling pathway, HR E6 proteins also activates telomerase reverse transcriptase to promote immortalization of cells [2].

Cdt2/DTL (denticleless) is an essential mammalian protein which also acts as a substrate adaptor for CRL4 cullin RING E3 ligase system. CRL_4_^Cdt2^ functions as one of the master regulators of cell cycle progression and genome stability [8]. It ensures the timely degradation of various cell cycle factors like p21, Set 8 and Cdt1 etc., which are involved in DNA replication initiation, progression, apoptotic checkpoint regulation and chromatin modification [9–11], in a replication-coupled and PCNA-dependent manner during S phase or after DNA damage [12]. The Cdt2 protein level is maintained in the cell by factors like CRL1^FBXO11^, CRL4^DDB2^, APC/C-Cdh1 system etc., which leads to its increase during G1 to S phase transition and then decreases during mitosis [13]. Cdt2 has been reported to be highly amplified in various cancers like lung cancer, breast cancer, colon cancer, erwig sarcoma, cervical cancer etc. [13]. It has been shown earlier in HR HPV cancer cells that E6 protein protects Cdt2 from ubiquitin-mediated degradation by recruiting a deubiquitinase, USP46 to deubiquitinate and stabilise Cdt2, which is essential for proliferation and survival of these cells [14].

MiRNAs (microRNAs) are small non-coding regulatory RNAs, 17–25 nucleotide long, which modulates the expression of more than 60% of genes at the post-transcriptional level in mammals [15]. They either degrade mRNA or inhibits translation by predominantly targeting 3’UTR (UnTranslated Region) sequences of mRNA complementary to its functional 8-base seed sequence [16]. In human genome, ~ 416 miRNA genes encode for ~ 340 distinct mature miRNAs known to date which can be either be a tumor promoter or suppressor based on its target mRNA. However, the molecular targets and the mechanism behind their functions for most of the miRNAs are yet to be discovered. Mutation or deletion in miRNA genes and their aberrant expression have been implicated in many diseases including cancers but the mechanism behind is poorly understood. Among these miRNAs, miR-34a which is located on chromosome 1p36 is believed to be a tumor suppressor and has been reported to be downregulated in several cancers, including cervical cancer [17–21]. Earlier it has been shown that p53 positively regulates miR-34a to induce apotosis [22]. However, the pathological and clinical relevance of miR-34a in cervical cancer is not well understood. In this study, we have explored the role of miR-34a in cervical cancer cell lines and discovered that Cdt2 is a novel target of miR-34a in HPV positive cervical cancer cells. It regulates Cdt2 expression by targeting E6, which earlier was shown to be responsible for stabilization of Cdt2 in HR HPV infected cervical cancer cells. miR-34a expression selectively affects the proliferation of these cancer cells, while non-cancerous cells don’t get affected much. To the best of our knowledge, this is the first ever report of miR-34a destabilizing Cdt2 by inversely regulating viral E6 protein. Hence, this study could pave the way for future research towards exploring the role of miR-34a as an alternate therapy for cervical cancer.

## Materials and methods

### Cell lines and media

HPV positive cervical cancer cell lines; SiHa and HeLa were purchased from the American Type Culture Collection (ATCC, USA) and HPV negative cervical cancer cell line; C33A and human kidney cell line HEK293T were purchased from the National Centre for Cell Science (NCCS, Pune, India). Unless mentioned, the cells were grown in Dulbecco’s Eagle Modified Medium (DMEM, Gibco, USA) supplemented with 10% Fetal Bovine Serum (FBS, Gibco, Brazil) and 1% Pen-Strep (Penicillin-Streptomycin, Gibco, USA). The cells were cultured at 37 °C, with 95% humidity and 5% CO_2_.

### Plasmids and miRNA

Empty plasmid backbone pcDNA 3.1 (used as control) was acquired from Addgene (Watertown, USA) and miR-34a plasmid was gifted by Moshe Oren (The Weizmann Institute of Science, Israel). Flag-Cdt2 and 16E6 plasmids were gifted by Dutta’s Lab (University of Virginia, USA). Other microRNA mimics were synthesized and purchased from Dharmacon (USA).

### Cell transfection

HEK293T, C33A, HeLa and SiHa cells were seeded (1X10^5^) in 6 well plates 24 hours prior to the transfection. The plasmid vectors (miR-34a, Flag-Cdt2 and 16E6) were transfected to the cells with turbofect™ (Thermo Fisher Scientific, Massachusetts, USA) the following day according to the manufacturer’s protocol. The cells were cultured at 37 °C, with 95% humidity and 5% CO_2_ for 48 hours.

### Western blot analysis

Cells were harvested and total protein was extracted using radioimmunoprecipitation assay (RIPA) buffer (50 mM Tris-Cl pH 7.5, 150 mM NaCl, 1% Nonidet P-40, 0.5% sodium deoxycholate,.1% sodium dodecyl sulfate, 1 mM PMSF) supplemented with protease inhibitor cocktail (Cell Signaling Technology, Massachusetts, USA). The protein concentration was measured using bradford protein assay (Sigma, Missouri, USA). The total protein extract was resolved on SDS-PAGE and then transferred onto either 0.22 μm or 0.45 μm PVDF membrane (Millipore, Massachusetts, USA). After the transfer, using protein ladder as reference, the membranes were cut according to the protein of interest as shown in the results (also refer to the raw images provided in the supplementary), then blocked with either 5% BSA (SRL, Mumbai, India) or 5% fat-free milk in TBST (Amersham, GE, Chicago, USA) for 1 hour at 4 °C followed by 1 hour at room temperature with gentle shaking. The membrane was then washed and probed with primary antibody overnight as per manufacturers’ instructions followed by washing and then incubating with secondary antibody (in TBST with 2.5% fat-free milk). The blot was then developed by enhanced chemiluminescence (ECL) substrate (Bio-Rad, California, USA) and documented using the chemidoc (Bio-Rad XRS+).

### Cell migration and cell invasion assays

Post 48 hours of transfection, cells were washed with PBS and resuspended in serum-free DMEM at 5X10^5^ cells/ml. For invasion assay, 1X10^5^ cells were placed in upper chamber of transwell plate (Corning, New York, USA), precoated with 2 mg/ml ECM gel (Sigma-Aldrich, Missouri, USA) and DMEM supplemented with 10% fetal bovine serum was added to the lower chamber. Cells were incubated in CO_2_ incubator for 48 hours. The remaining cells from upper chamber were removed and the cells invaded through the membrane were fixed with 5% glutaraldehyde. Invaded cells were stained with 0.2% crystal violet solution in 2% ethanol and counted under inverted microscope.

For migration assay, 1X10^5^ cells were placed in upper chamber of the transwell plate and DMEM supplemented with 0.5% FBS and 40 μg/ml collagen I (Sigma-Aldrich, Missouri, USA) was added to the lower chamber. Cells were incubated for 24 hours in CO_2_ incubator. The remaining cells were removed from upper chamber and the migrated cells were fixed with 5% glutaraldehyde. The migrated cells were stained with 0.2% crystal violet solution in 2% ethanol and counted under inverted microscope.

### Immunofluorescence

Post 48 hours of transfection, 1X10^4^ cells were plated on glass coverslips placed in 6 well plate and incubated with DMEM medium containing 10% FBS for 24 hours in CO_2_ incubator. Cells were then washed with PBS and fixed with 4% paraformaldehyde in PBS for 20 mins. Fixed cells were permeabilized with 0.1% Triton X-100 in PBST and blocked with 5% BSA for 1 hour. Coverslips were incubated with primary antibody overnight at 4 °C in dark followed by incubation with secondary antibody at 4 °C in dark humid chamber for 1 hour. The coverslip was then mounted on slide using mounting media (Vectashield, Vector Laboratories, San Francisco) and observed under confocal microscope (STED, Leica, Wetzlar, Germany).

### Statistical analysis

All the western experiments were performed in biological triplicates. The immunofluorescence, growth curve, migration and invasion assays were performed in experimental triplicates. All the data are presented as the mean ± SD and an unpaired student’s t-test was performed to calculate the significant value. Image J software was used to quantify the intensity of protein bands wherever applicable.

## Results

### miR-34a destabilizes Cdt2 protein in HPV positive cervical cancer cells

**Fig. 1:**
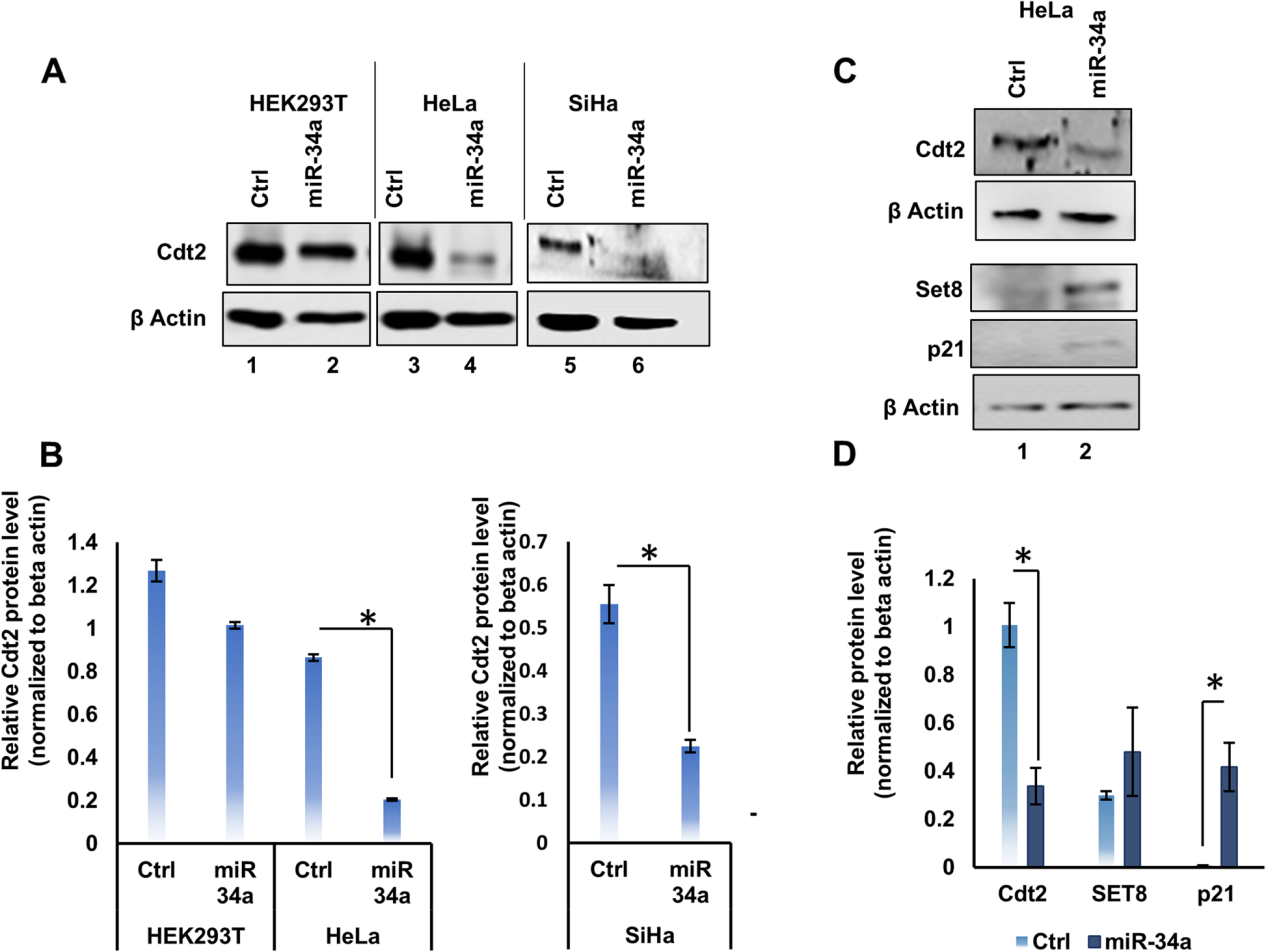
Overexpression of miR-34a decreases Cdt2 expression level in cervical cancer cells. **A** Western blot analysis was performed for Cdt2 protein level upon 48 h treatment with miR-34a in HPV positive cells (HeLa and SiHa) and HEK293T cells. **B** Quantification of the Cdt2 protein level in HEK293T, HeLa and SiHa cell lines upon transfection with miR-34a for 48 h in comparison to their respective vector control. **C** Western blot analysis was performed for p21 and Set8 protein levels upon 48 h treatment with miR-34a in HPV positive cells (HeLa cells) in comparison to the cells transfected with control. **D** Quantification of SET 8 and p21 protein level in HPV positive cell line upon transfection with miR-34a for 48 h in comparison to vector control. All the experiments representing the quantitative values were done in triplicates. Error bar represents S.D. * represents significant difference as compared with control at *p* value < 0.05

Previous studies have shown that in HR HPV infected cervical cancer cells, Cdt2 is stabilized by HPV E6 protein [14]. Also, it has been well established that miR-34a is downregulated in cervical cancers [21]. In order to investigate whether there is a correlation between miR-34a and expression of Cdt2, we transfected HEK293T cells (non-cancerous cell line) and two HR HPV positive cervical cancer cell lines SiHa and HeLa with miR-34a. A significant reduction in expression of Cdt2 was detected in both the cervical cancer cell lines (Fig. 1A Lane 3, 4, 5 and 6 and Fig. 1B), whereas ectopic expression of miR-34a did not cause any significant change in expression of Cdt2 in HEK293T cell lines (Fig. 1A Lane 1and 2 and Fig. 1B).

Since Cdt2 facilitates degradation of p21 and Set 8 [8, 14], we next checked the level of p21 and Set 8 upon miR-34a expression in HR HPV positive cervical cancer cells and found that Cdt2 destabilization stabilizes both p21 and Set 8 protein level significantly (Fig. 1C and D).

### miR-34a destabilizes Cdt2 in cervical cancer cells by targeting HPV protein E6

**Fig. 2:**
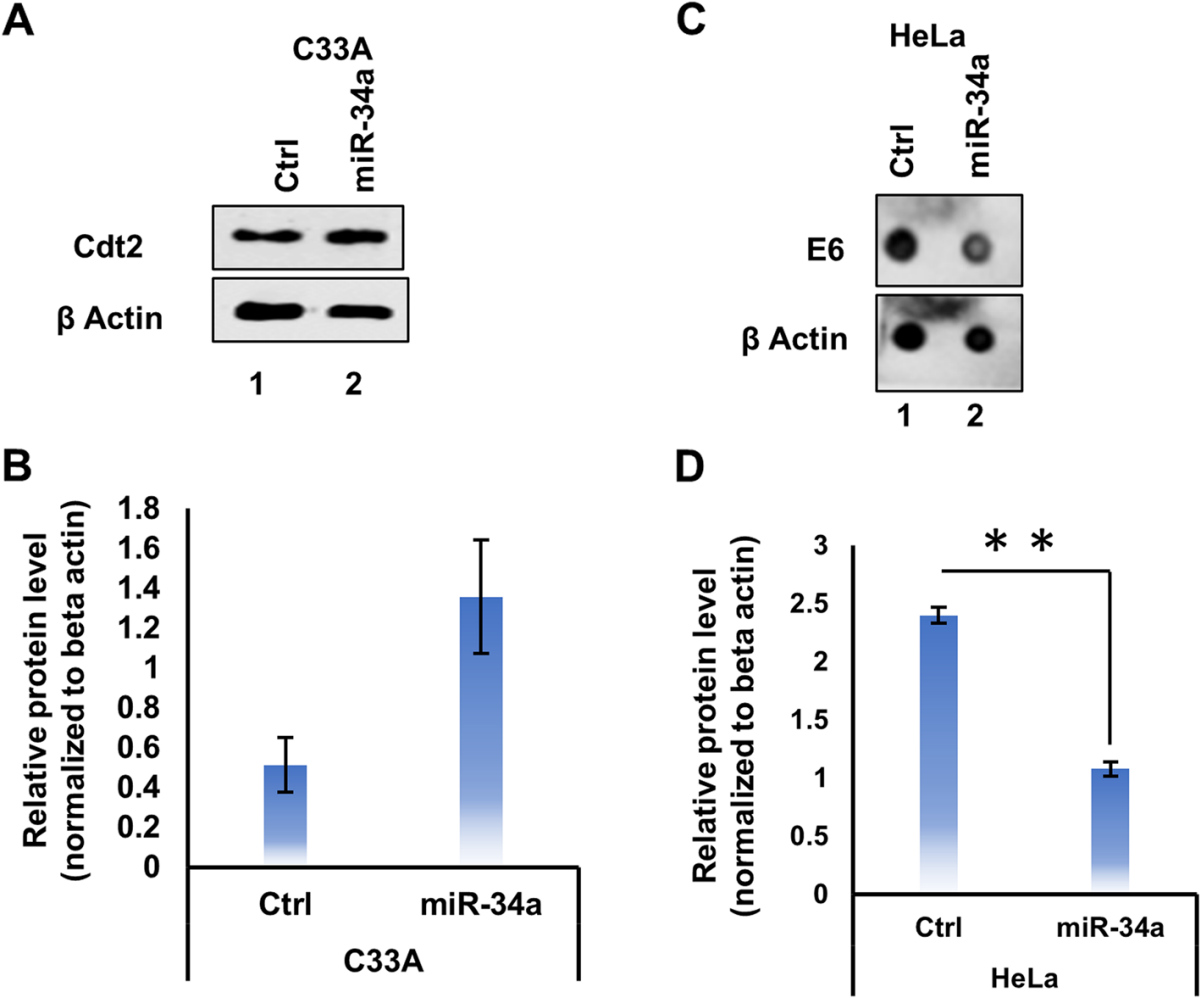
MiR-34a decreases Cdt2 expression in HPV infected cervical cancer cells by targeting HPV E6. **A** Western blot analysis was performed for Cdt2 protein level upon 48 h treatment with miR-34a in HPV negative cervical cancer cells (C33A). **B** Quantification of the Cdt2 protein level in C33A cell line upon transfection with miR-34a for 48 h in comparison to vector control. **C** Dot blot analysis was performed for E6 protein level upon 48 h after transfection in HPV positive cell (HeLa) with miR-34a in comparison to relative control. **D** Quantification of E6 protein level in HPV positive cell line (HeLa) upon transfection with miR-34a for 48 h in comparison to vector control. All the experiments representing the quantitative values were done in triplicates. Error bar represents S.D. ** represents significant difference as compared with control at *p* value < 0.001

We have shown that miR-34a destabilizes Cdt2 level in HPV positive cervical cancer cells but does it also destabilize Cdt2 in HPV negative cervical cancer cells? To answer this question, we transfected C33A (non-HPV cervical cancer) cells with miR-34a expressing plasmid and surprisingly discovered that miR-34a expression does not suppress Cdt2 level in the C33A cell line (rather it slightly increases Cdt2 [non-significant], Fig. 2A and B).

Since E6 is the major regulator in HPV infected cells, we wanted to investigate the effect of miR-34a on E6 expression. The dot blot profile of E6 protein in the HeLa cells shows that miR-34a expression reduces the E6 protein level significantly (Fig. 2C and D), suggesting that miR-34a/Cdt2 axis is directly regulated by E6 suppression in cervical cancer.

### miR-34a suppresses the proliferation of HPV infected cervical cancer cells

**Fig. 3:**
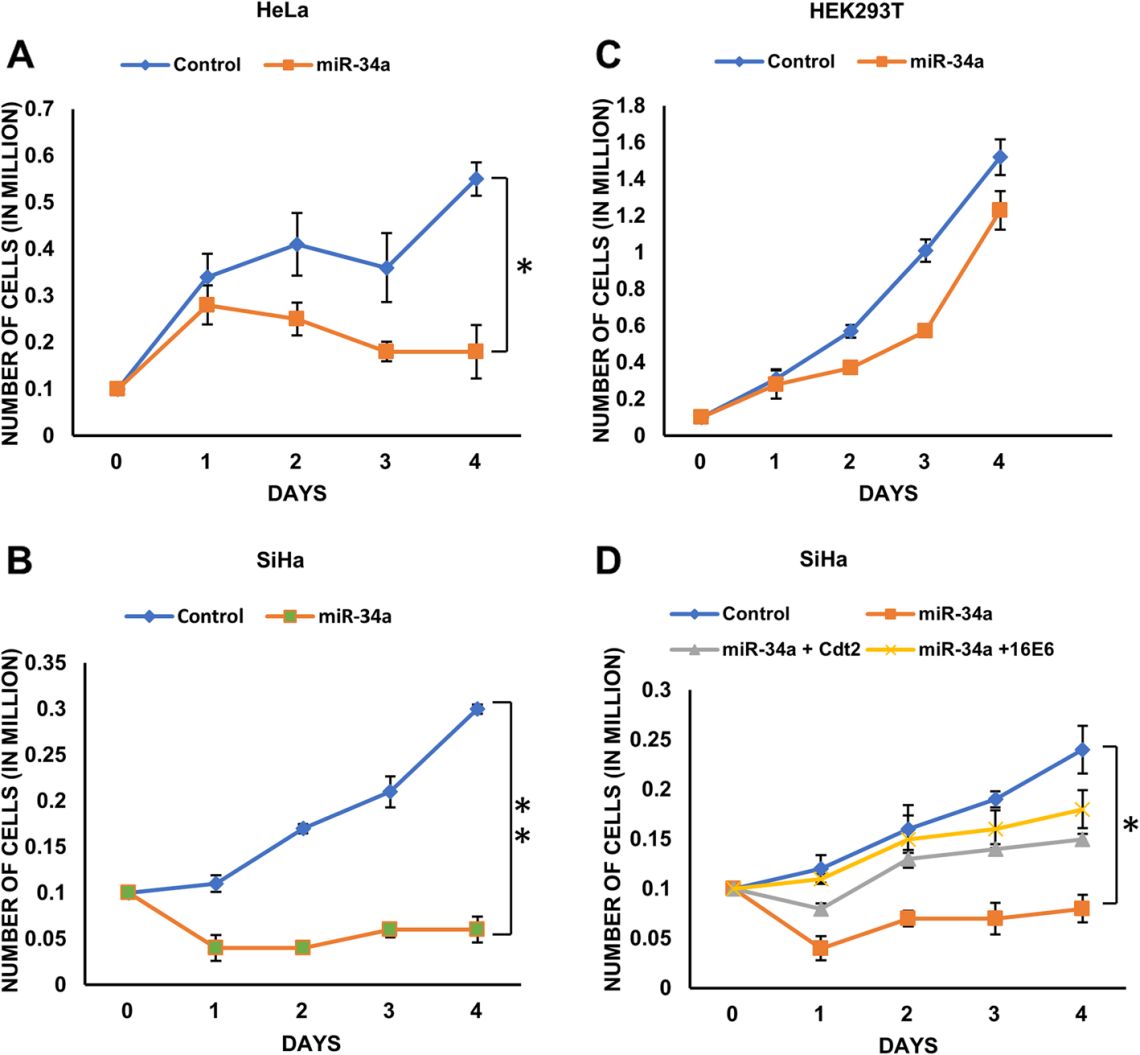
Ectopic expression of miR-34a suppresses cell proliferation in HPV positive cervical cancer cells. **A** Growth curve for HeLa-miR-34a shows the reduction in growth in comparison to relative control. **B** Growth curve for SiHa-miR-34a shows the reduction in growth in comparison to relative control. **C** Growth curve for HEK293T-miR-34a shows the growth inhibition which is compensated by day 4. **D** 0.1 million SiHa cells were seeded 24 hours prior to transfection with mock, miR-34a, miR-34a + Cdt2 and miR-34a + 16E6 and cell growth were observed every 24 hours for 4 days. Each value represents the mean of three readings. Error bar represents S.D. * and ** represents significant difference as compared with control at *p* value < 0.05 and *p* value< 0.001 respectively

We have already shown that miR-34a destabilizes Cdt2 and E6 which are one of the major players of proliferation and tumor growth in HR HPV infected cervical cancer cell lines. Therefore, we wanted to see what role miR-34a overexpression plays in growth and proliferation of cervical cancer cells. We observed that mir-34a expression significantly inhibits the growth of cervical cancer cell lines (Fig. 3A and B). While in case of HEK293T cells, miR-34a overexpression decreases the cell proliferation initially (at 48 hours) which get normalized by 72 hours (Fig. 3C).

Further, we wanted to see if decreased proliferation rate of HPV positive cells upon miR-34a expression could be normalized by overexpression of Cdt2 or E6 proteins. For this, we transfected SiHa cells with either miR-34a alone or miR-34a in combination with 16E6 plasmid or miR-34a in combination with Flag-Cdt2 plasmid. The results showed that ectopic expression of 16E6 or Cdt2 could rescue the growth of cervical cancer cells (Fig. 3D). However, the rescuing of inhibited proliferation is more in case of miR-34a and 16E6 co-transfection compared to miR-34a and Flag-Cdt2 co-transfection. We had also co-transfected SiHa cells with all the three vectors containing miR-34a, Flag-Cdt2 and 16E6 but it proved to be toxic and could not rescue the growth inhibition caused by miR-34a expression (Fig. S1).

### miR-34a expression causes morphology change and cell growth suppression

**Fig. 4:**
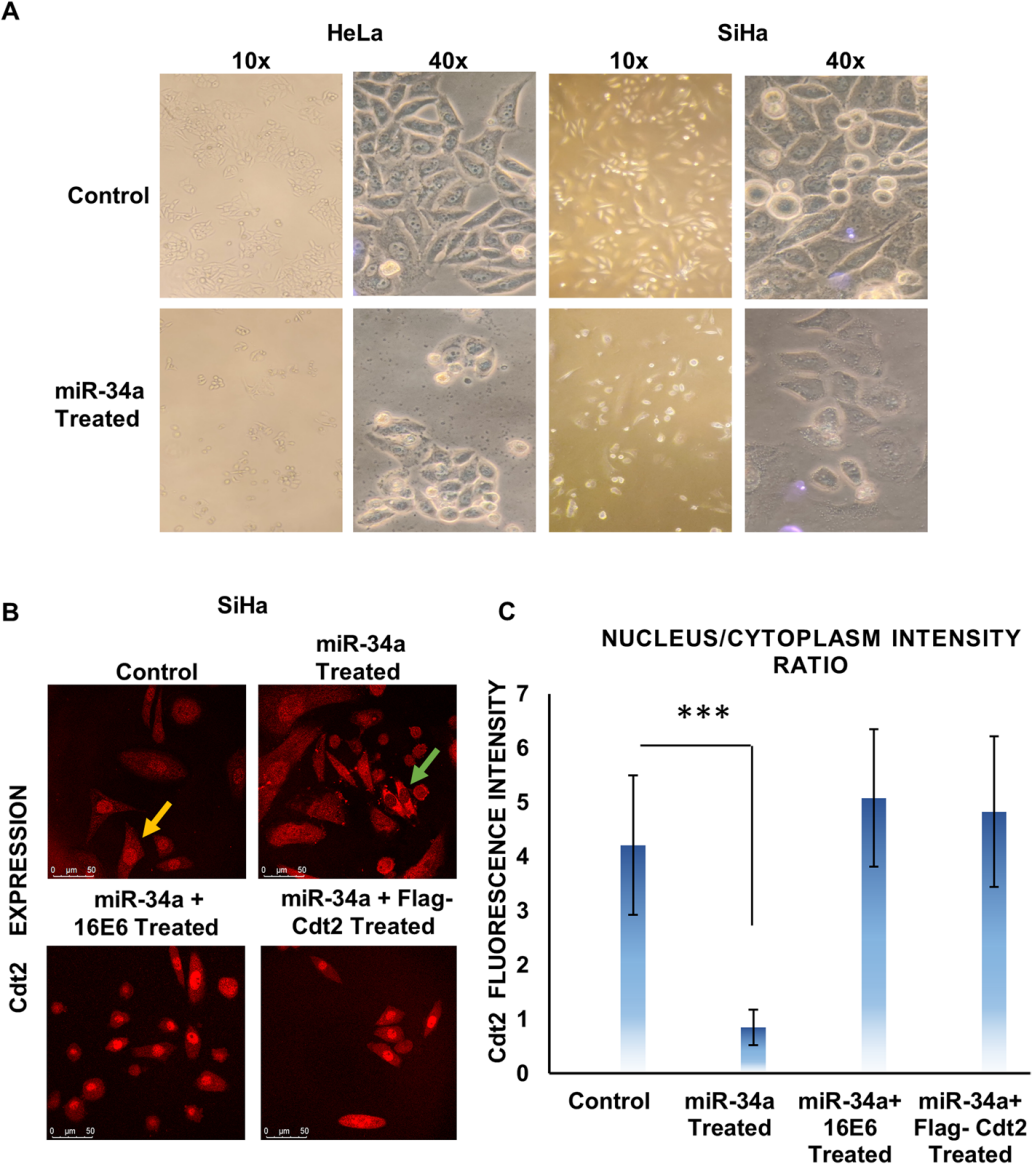
MiR-34a expression causes morphology change. **A** HeLa and SiHa cells were transfected with miR-34a for 48 hours and phase-contrast microscopy was performed at 10X and 40X magnifications. **B** SiHa cells were transfected with miR-34a, miR-34a + Cdt2 and miR-34a + 16E6 for 48 hours, probed with anti-Cdt2 proteins and confocal microscopy was performed. Scale bar, 50 μm. **C** Quantification Graph for Cdt2 nucleus/cytoplasm intensity ratio. Results are shown as mean of 20 different randomly selected cells and Cdt2 intensity level in nucleus/cytoplasm were quantified. Error bar represents S.D. *** represents *p* value < 0.0001

To observe the morphological changes occurring in the cervical cancer cells upon miR-34a transfection we performed phase-contrast microscopy and observed that the untreated HeLa and SiHa cells maintained their morphology and were in close contact with each other even after 48 hours of incubation. In contrast, the miR-34a treated cells showed deformed morphology after 48 hours of treatment (Fig. 4A magnification 40X). Also, miR-34a transfected HeLa and SiHa cells have lower confluency than the control (Fig. 4A), suggesting decreased proliferation rate.

Cdt2 is involved in G1 to S phase transition of the cell cycle and is majorly localized in the nucleus [23]. By confocal microscopy, it was evident that Cdt2 is enriched in the nucleus of SiHa cells as indicated by yellow arrow in Fig. 4B (control, Cdt2 nucleus/cytoplasm intensity ratio of 4.21, Fig. 4C) but get exported to the cytoplasm upon miR-34a overexpression (Cdt2 nucleus/cytoplasm intensity ratio 0.85, green arrow Fig. 4B and C). Also, the miR-34a treated HPV positive cervical cancer cells showed lower confluency (higher number of dead cells). Interestingly, co- transfection of miR-34a with either 16E6 or Cdt2 (Flag-Cdt2) restores back the Cdt2 level in the nucleus and also increases the confluency of the cells (Figs. 3D, 4B, and C). This explains the rescue from growth suppression upon co-transfection (Fig. 3D).

### Upregulation of miR-34a suppresses both cell migration and invasion of HPV infected cervical cancer cell lines

**Fig. 5:**
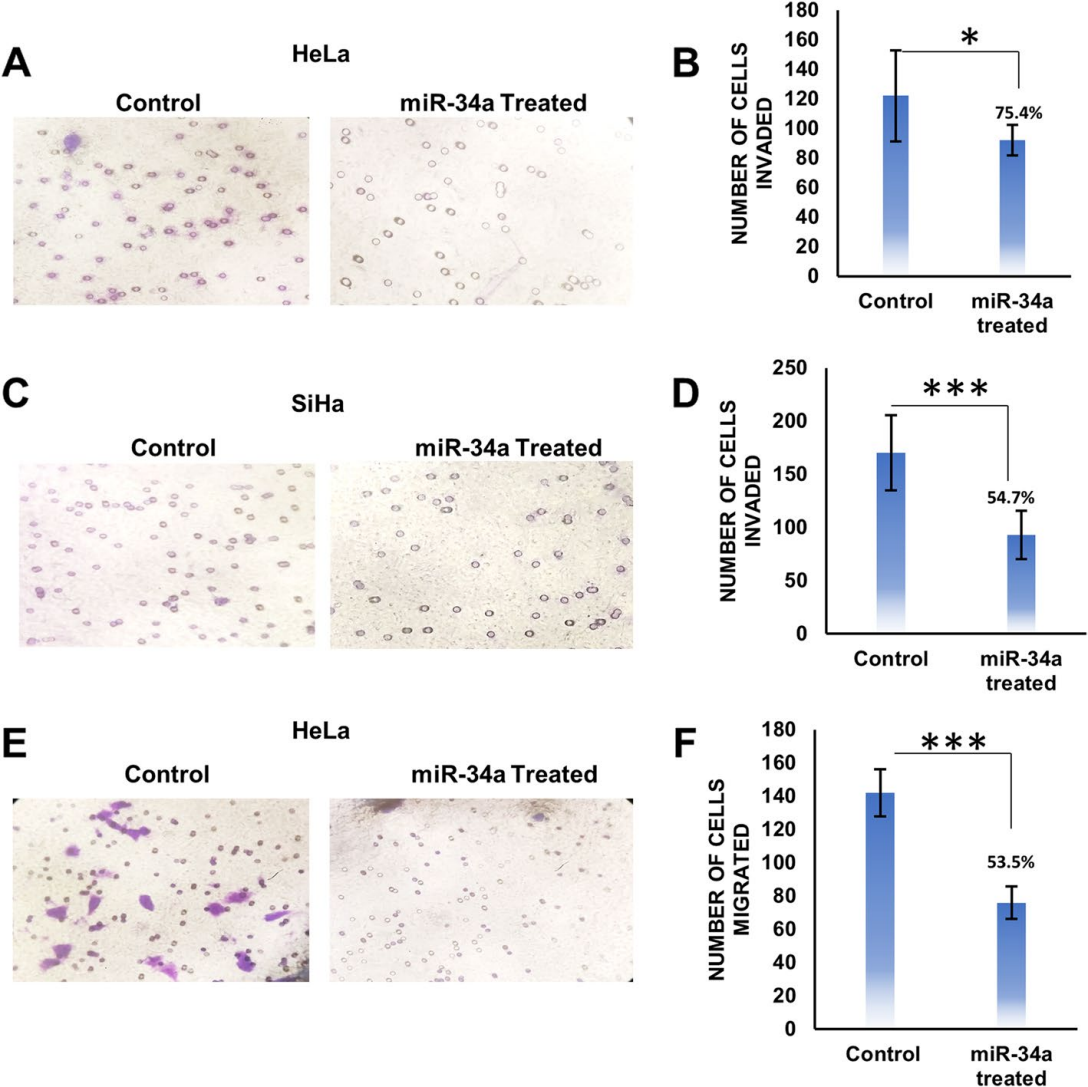
Effect of miR-34a expression on transwell migration and invasion. **A** Representative images of transwell invasion assay of HeLa cells with ectopic expression of miR-34a and respective vector control. **B** Graphical representation of transwell invasion assay of HeLa cells with ectopic expression of miR-34a (92 ± 10.38), showing the reduction in transwell invasion in comparison to vector control (122 ± 30.64). **C** Representative images of transwell invasion assay of SiHa cells with ectopic expression of miR-34a and respective vector control. **D** Graphical representation of transwell invasion assay of SiHa cells with ectopic expression of miR-34a (93 ± 22.74), showing the reduction in transwell invasion in comparison to vector control (170 ± 35.40). **E** Representative images of transwell migration assay of HeLa cells with ectopic expression of miR-34a and respective vector control. **F** Graphical representation of transwell migration assay of HeLa cells with ectopic expression of miR-34a (76 ± 9.66), showing the reduction in transwell migration in comparison to vector control (142 ± 14.29). 8 different areas were selected randomly and number of invaded and migrated cells were counted. Error bar represents S.D. * represents *p* value < 0.05, *** represents *p* value < 0.0001

In order to determine what role miR-34a plays in cell proliferation, metastasis and invasion of HPV positive cervical cancer cells, trans-well invasion and migration assays were performed. The invasive potential of HeLa and SiHa cells transfected with miR-34a gets reduced by 24.6 and 45.3% respectively, in comparison to control transfected cells (Fig. 5A-D). We also observed that the migration level of HeLa cells was decreased by ~ 0.5-fold after transfection with miR-34a, compared to control (Fig. 5E and F). Hence, both invasion and migration were significantly suppressed by overexpression of miR-34a.

## Discussion and conclusion

Dysregulation of miRNAs has been linked with various cancers [24, 25]; these biomolecules can act either as tumor suppressors or tumor promoters [26, 27]. Cervical cancer which is one of the deadliest cancers in women worldwide (fourth-largest killer among cancers) is caused by high-risk HPV infection which interfering with many cellular processes, drives the cells towards oncogenic transformation. Several miRNAs have been reported to be mis regulated in cervical cancer cells (both in patient samples and cell lines data [28–30];) and one of them is miR-34a, which is encoded on chromosome 1p36 [17, 19] in humans. miR-34a has been shown to play a critical role in maintaining the healthy functioning of cells by acting as a tumor suppressor in many solid tumors. It has been reported to repress over 700 transcripts involved in cellular proliferation, survival and plasticity [31], however, very little is known about the mechanisms involved in this process.

In this study, we have discussed the mechanistic role of miR-34a in regulation of an essential cell cycle factor Cdt2/DTL which is involved in G1 to S phase transition [8, 32, 33]. We have shown that miR-34a destabilizes Cdt2 in HPV positive cervical cancer cells (Fig. 1) by suppressing viral E6 protein (Fig. 2C). This is the first-ever study to show that HR HPV E6 oncoprotein, which suppresses many tumor inhibitor proteins, is directly regulated by a micro-RNA, miR-34a.

E6 oncoprotein of HR HPV hijacks many cell cycle pathways and prevents the host cell from going in cell cycle arrest and apoptosis in response to the genotoxic viral infection [2, 14]. It has been shown earlier that, HPV E6 protein stabilizes Cdt2 by recruiting a deubiquinitase USP46 inside the nucleus, which in turn proves to be essential for proliferation and survival of the HR HPV infected cancerous cells both in-vitro and in-vivo [14]. Cdt2 is an adaptor protein of CRL4 E3 ubiquitin ligase complex and is involved in proteasomal degradation of cell cycle factors like p21, Cdt1, Set8 etc., for the progression of G1/S and S phase to the M phase [8]. Silencing of E6 gene (by siRNA) has been shown to exclude USP46 from the nucleus, which in turn promote polyubiquitination mediated degradation of Cdt2, eventually leading to apoptosis and cell death of these cancer cells [14].

In this study, we show that destabilization of Cdt2 by miR-34a is specific to HPV positive cervical cancer cell lines (Fig. 1A Lane 3, 4, 5, 6 and 1B) while not much change is observed in non-cervical cancer cells (Fig. 1A lane 1, 2 and 1B) or HPV negative cervical cancer cells (Fig. 2A and B), indicating that Cdt2 is not a direct target of miR-34a. Since, Cdt2 is a major component of CRL4-E3 ubiquitin ligase system, destabilization of Cdt2 leads to upregulation of proapoptotic and onco-suppressor proteins p21 and Set8, which otherwise is present in very low levels in HPV positive cervical cancer cells (Fig. 1C and D). In earlier studies, ectopic expression of miR-34a has been shown to arrest the cell cycle at G1/S and G2/M phase (by FACS analysis of DNA content) in cervical cancer cells [34]. Our results explain the above finding that it might be due to the elevated levels of p21 and Set8 proteins [8, 35].

Further, to the best of our knowledge, for the first time our finding shows that miR-34a overexpression decreases the levels of HPV E6 protein (Fig. 2C and D), which earlier was shown to deubiquitinate and stabilize Cdt2 protein by importing a deubiquitinase USP46 from cytoplasm to nucleus [14]. Hence, suppression of E6 by miR-34a provides favorable condition for CRL1^FBXO11^ or CRL4^DDB2^ E3 ligase mediated ubiquitination and degradation of Cdt2. By now, it has been well established through various reports that miRNA by binding to the 3′ UTR sequence can degrade or inhibit the target mRNA in mammalian cells [36] and our result shows that viral E6 is a target of miR-34a in HPV infected cervical cancer cell lines.

It has been shown earlier that cell cycle factor Cdt2 is essentially required for hyperproliferation and survival of HPV positive cervical cancer cells, therefore destabilization of E6 by miR-34a reduces the level of Cdt2 which critically slows down growth and proliferation of HPV positive cervical cancer cells (Figs. 3A, B and 4A 10x magnification). While, in the non-cancerous cell line (HEK293T) there is negligible suppression of Cdt2 upon overexpression of miR-34a, and the proliferation rate catches up within 72 hours to normalcy (Fig. 3C). Change in cellular morphology could be seen in miR-34a overexpressed HPV positive cancerous cells (Fig. 4A) indicating that Cdt2 suppression is causing cellular senescence probably due to halted G1 to S phase transition [14], while no such change was observed in non-cancerous cells (Fig. S2). This suppression in proliferation of HPV positive cervical cancer cell lines can be compensated by overexpression of either Cdt2 or E6 (Fig. 3D), indicating inverse relation between the miR-34a expression and cervical cancer cell proliferation. Also, we have shown in this study that ectopic expression of viral E6 or Cdt2 along with miR-34a restores Cdt2 levels in the nucleus which was otherwise excluded from nucleus upon miR-34a overexpression (Fig. 4B) due to suppression of E6 protein. This explains the rescue of growth upon co-transfection of miR-34a with either E6 or Cdt2 (Fig. 3D). Additionally, miR-34a overexpression inhibits the cell invasion and migration abilities of these cervical cancer cells (Fig. 5A-F) by inhibiting cell proliferation and inducing cell senescence [22, 37–40].

**Fig. 6:**
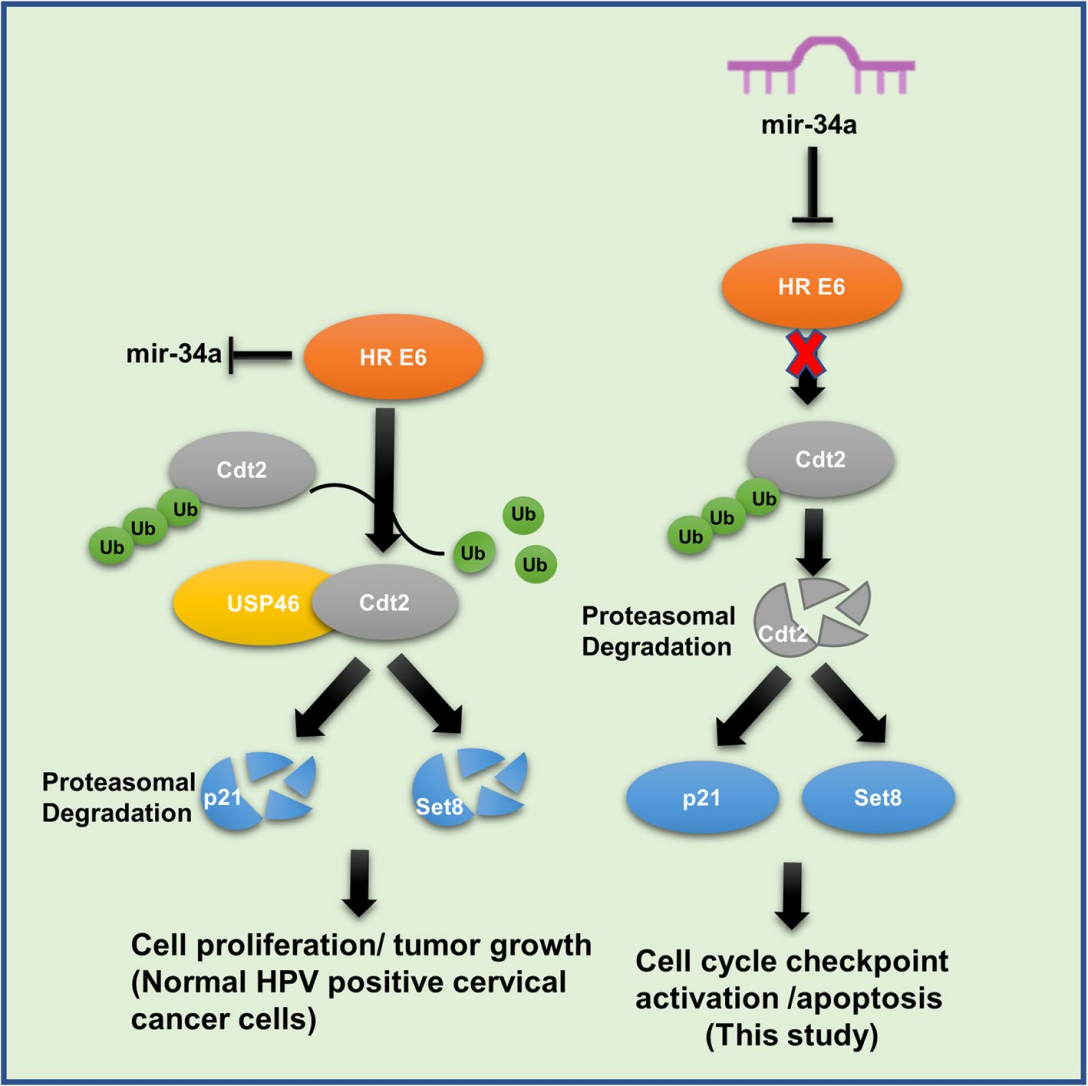
MiR-34a expression suppresses HPV E6 protein which destabilizes Cdt2, leading to stabilization of p21 and Set8, ultimately activating cell cycle checkpoints and apoptotic pathways

E6 is an oncogenic protein that either suppresses or leads to degradation of several tumor suppressor proteins and checkpoint systems, which in turn leads to transformation and hyperproliferation of HPV infected cells. It also promotes stability of oncogenic cell cycle factor like Cdt2, empowering re-replication and hyperproliferation. MiR-34a which happens to be a tumor suppressor miRNA, is downregulated in many cancers including cervical cancer. Our study is the first to show that an anti-tumorous microRNA, miR-34a suppresses viral E6 protein (which in turn excludes USP46 from nucleus) and destabilizes overexpressed oncoprotein Cdt2 in HPV positive cell lines and stops their proliferation eventually killing these cancerous cells (Fig. 6), while no effect is seen on HPV negative cells. Since 99% cervical cancers are due to high-risk HPV infection [2], our study opens up the potential of using miR-34a for HPV infected cervical cancer therapy which could be much cheaper, noninvasive and specific treatment plan for such fatal cancer.

## Supplementary Information

**Additional file 1.**

**Additional file 2.**

**Additional file 3.**

## Data Availability

All data generated or analyzed during this study are included in this article (and its supplementary information files and RAW PDF file) and also available from the corresponding author on reasonable request.
