# Supplementary material for: miR-34a negatively regulates cell cycle factor Cdt2/DTL in HPV infected cervical cancer cells
Source: BMC Cancer. 2022 Jul 15;22:777. doi: 10.1186/s12885-022-09879-5 (PMC9288023; doi:10.1186/s12885-022-09879-5)
Supplement: Supplementary file 1 — Additional file 1. [file 12885_2022_9879_MOESM1_ESM.pdf]

Raw file for Figure 1 A

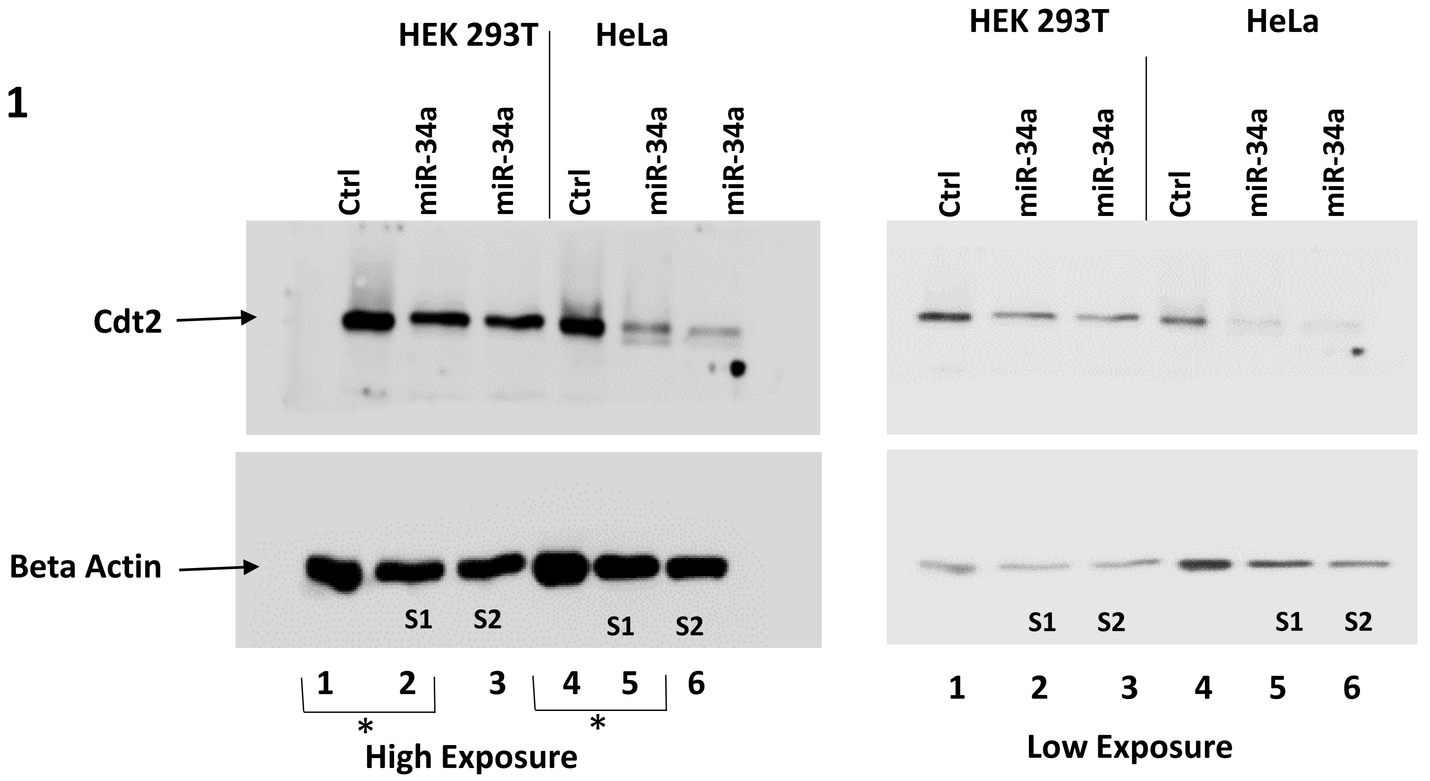

BLOT 1 → Represents original of the blot given in manuscript (both high and low exposure as mentioned above)
* Lane no. 1, 2 and 4, 5 have been shown in the manuscript respectively.

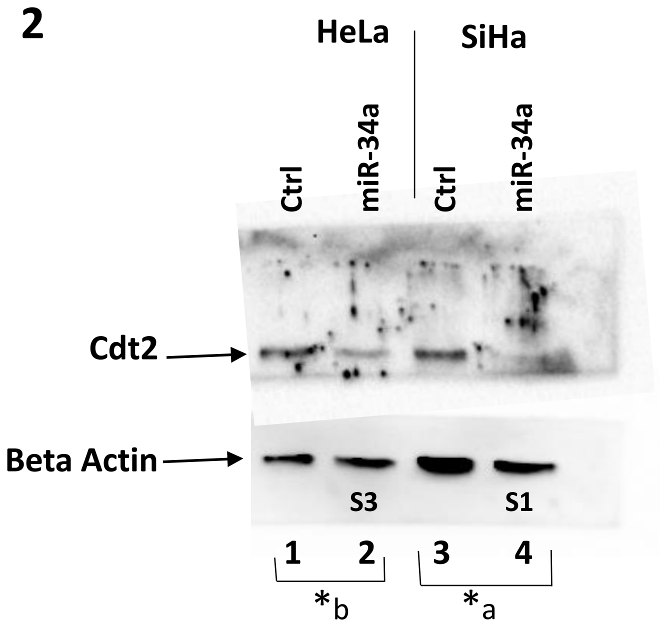

BLOT 2 → Represents original of the blot given in manuscript

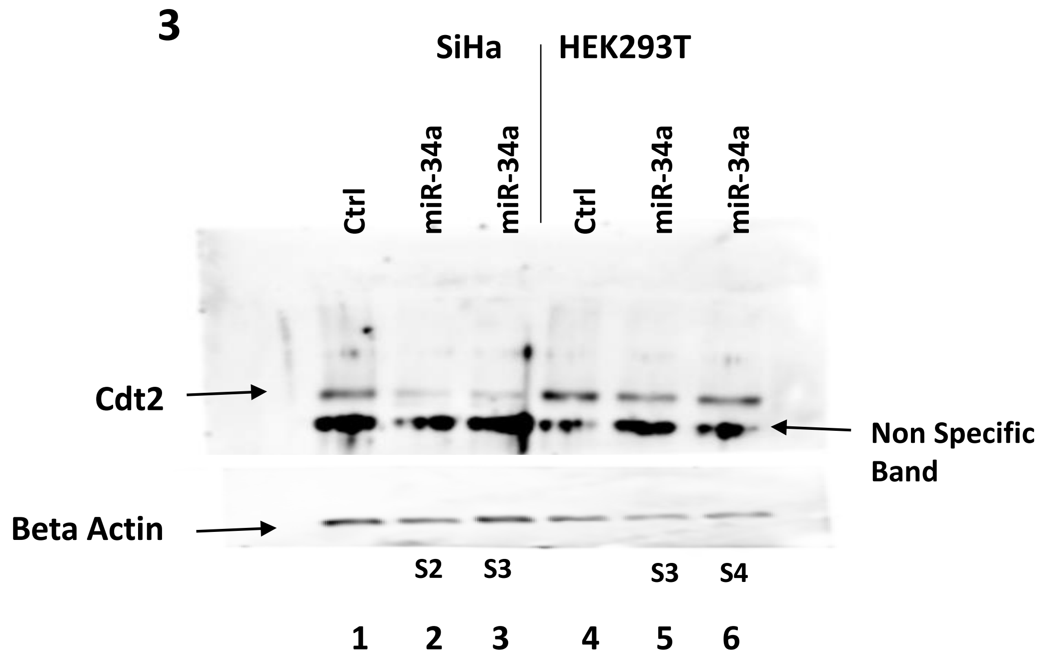

BLOT 3

***a:** Lane 3 and 4 represents original of the blot for SiHa given in Fig. 1A of the manuscript
***b:** Lane 1 and 2 represents original of the blot for HeLa given in Fig. 1C of the manuscript

Western blot followed by immunostaining was performed for Cdt2 protein level after 48h treatment with miR-34a in HEK293T, HeLa and SiHa cell lines. S1,S2,S3 and S4 represents the protein extracted from biological replicates of the treated samples respectively. **1:** represents **BLOT 1** showing Cdt2 protein level in HEK293T and HeLa cells (both at higher exposure and low exposure), **high exposure blot was cropped for lane 1,2 and 4,5 in original manuscript** . **2:** represents **BLOT 2** showing original blot for Cdt2 protein level in HeLa and **SiHa samples (from which Lane 3 and 4 was given in original manuscript**). **3:** represents **BLOT 3** showing Cdt2 protein level in SiHa and HEK293T samples.

Note: Sometimes middle portion (in between Cdt2 and Beta actin bands) of the blots were cut to use for immunoblotting different protein (for other unpublished experiment).

Raw file for Figure 1 C

←----------------HeLa--------------------→ ←----------------HeLa--------------------→

1

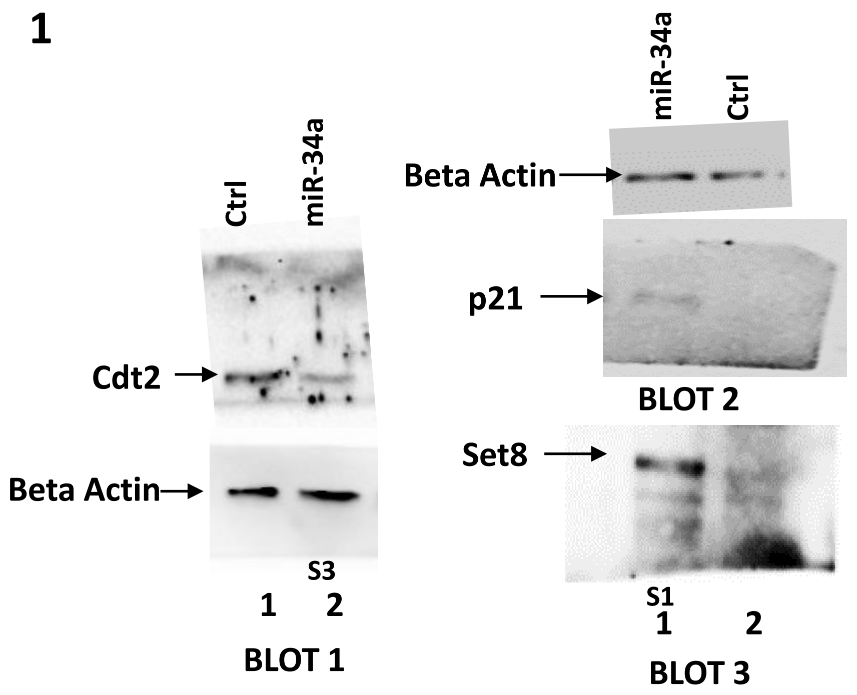

BLOT 1, 2 and 3→ Represents original of the blots given in manuscript for Fig.1C

2

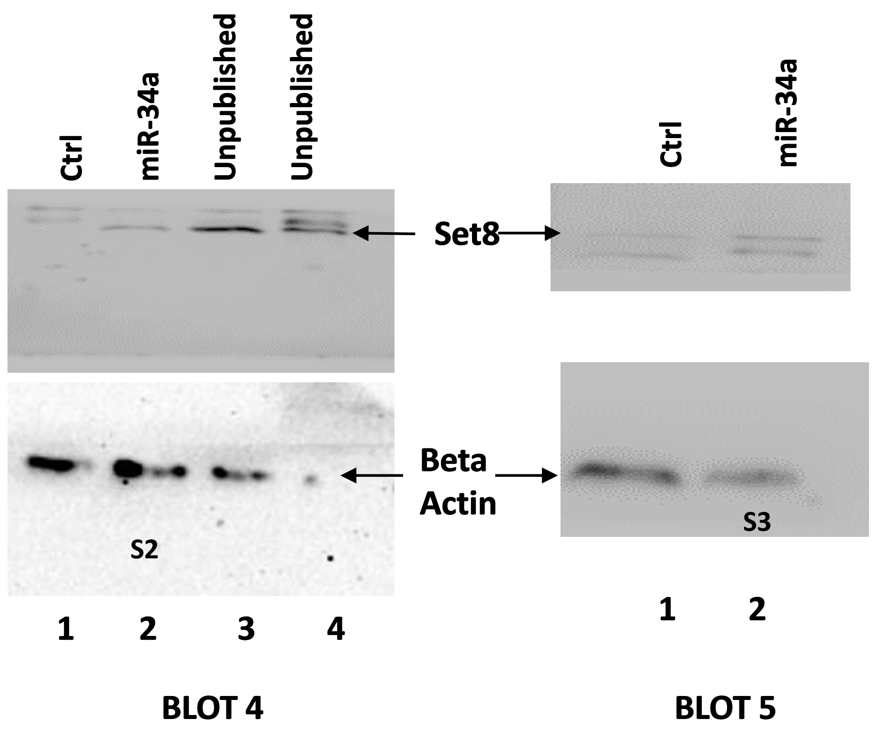

BLOT 4 and 5→Used as replicates for quantification of Set8

3

←--HeLa----------------------------------→

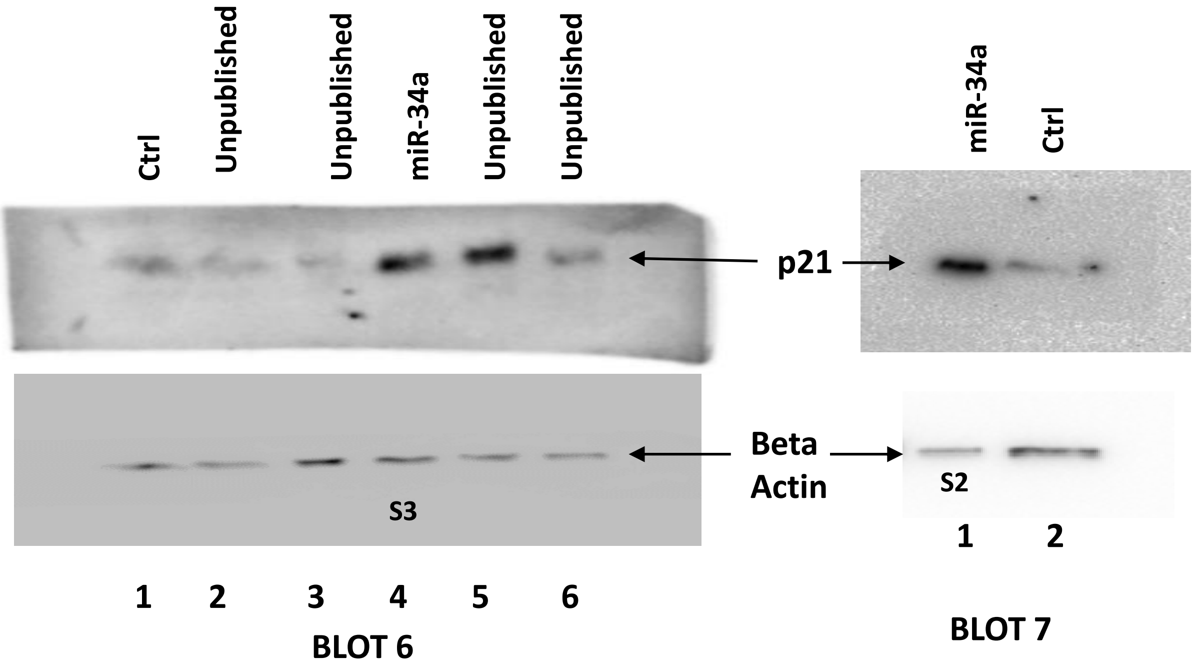

BLOT 6 and 7→ Used as replicates for quantification of p21

Western blot followed by immunostaining was performed for Cdt2, p21 and Set8 protein level after 48h treatment with miR-34a in HeLa cell line.S1,S2 and S3 represents the biological replicates of the treated samples which were used for quantification of the same. **1:** represents **BLOT 1,** corresponds to Cdt2 protein level from Fig. 1C. **BLOT 2** corresponds to p21 and beta actin protein level (given in manuscript). **BLOT 3** corresponds to Set8 protein level (given in manuscript). p21 and beta actin were processed in same blot while Set8 was processed parallelly on different blot because Set8 and Beta actin has overlapping molecular weights to each other. Samples for **BLOT 1, 2** and **3** were derived from same biological experiment. **2:** represents **BLOT 4** and **5** showing the replicates used for quantification of Set8 protein level in HeLa cells upon miR-34a treatment. **3:** represents **BLOT 6** and **7** showing the replicates used for quantification of p21 protein level in HeLa cells upon miR-34a treatment.

Note: BLOT 4 and **6** were developed during initial screening times when we were checking other microRNAs as well, hence the blot also contains other data which is labelled here as unpublished.

Here, we have only mentioned the data which is related to this study.

Raw File of Figure 2A

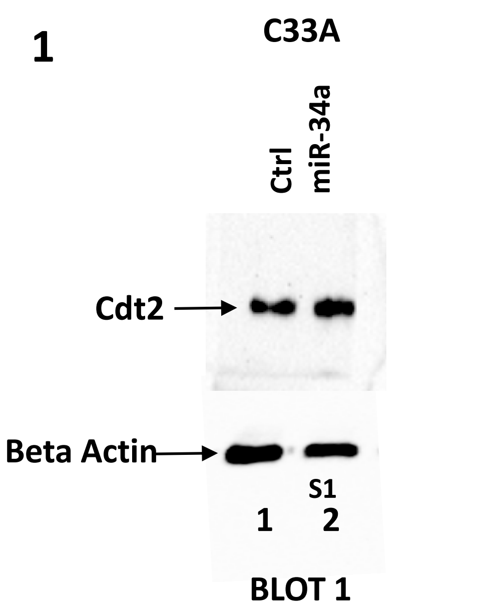

BLOT 1 → Represents original of the blot given in manuscript for Fig.2A

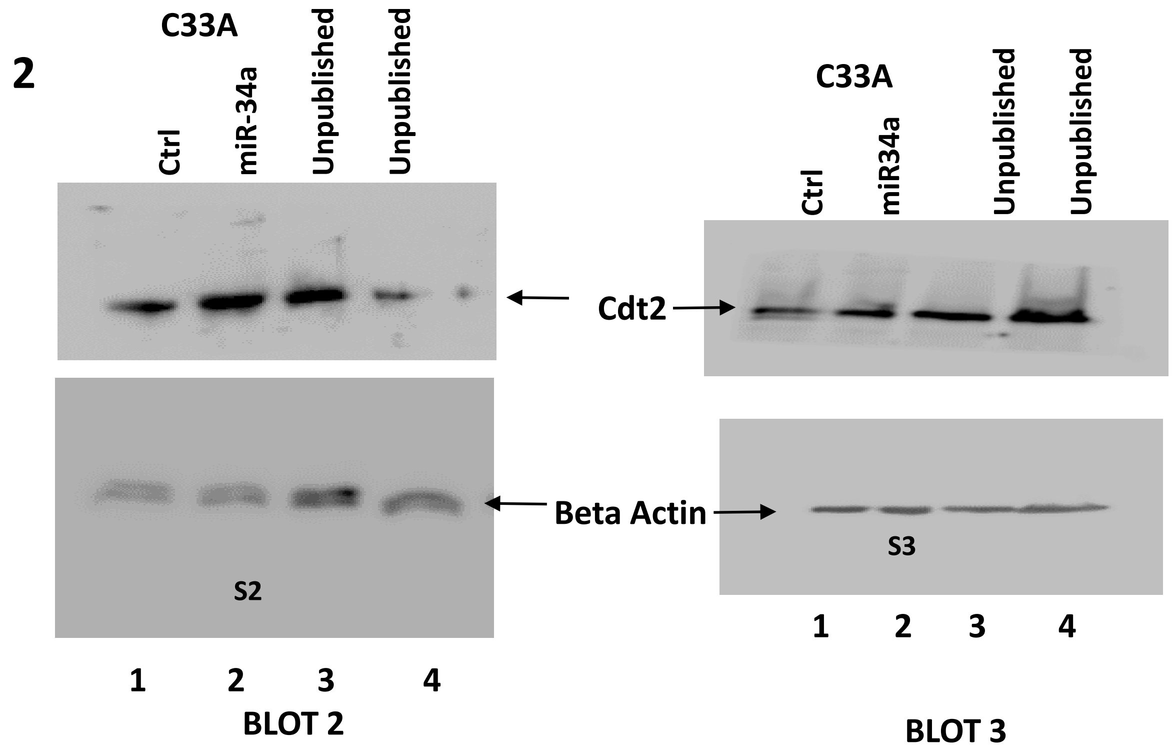

BLOT 2 and 3→Used as replicates for quantification of Cdt2

Western blot followed by immunostaining was performed for Cdt2 protein level after 48h treatment with miR-34a in HPV negative cervical cancer cells (C33A) which corresponds to Fig. 2A in main manuscript file. S1,S2 and S3 represents the biological replicates of the treated samples which were used for quantification of the same. **1:** represents **BLOT 1** showing Cdt2 protein level in C33A after miR-34a treatment given in original manuscript **2:** represents **BLOT 2** and **3** showing the biological replicates used for quantification of Cdt2 protein level in C33A after miR-34a treatment.

NOTE: BLOT 2 and **3** consist of data which is related to other study hence labelled here as unpublished. **Here, we have only mentioned the data which is related to this study only.**

Raw File of Figure 2C

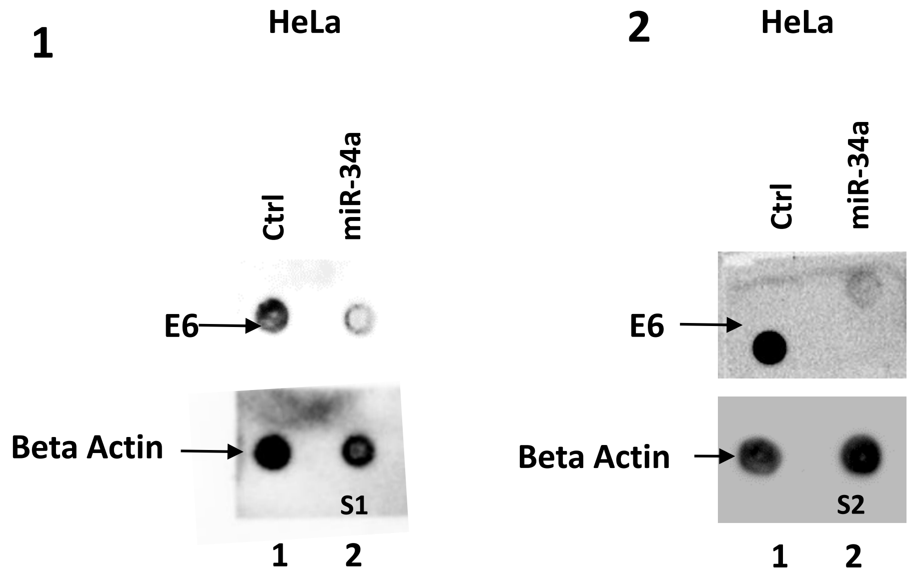

BLOT 1 → Represents original of the blot given in manuscript for Fig.2C

BLOT 2

Dot blot followed by immunostaining was performed for HPV E6 protein level after 48h treatment with miR-34a in HPV positive cervical cancer cells (HeLa) for Fig. 2C in the main manuscript file. S1 and S2 represents the biological replicates of the treated samples which were used for quantification of the same. **1 and 2:** Represents the **BLOT 1** and **2** showing E6 protein level in HeLa after miR-34a treatment.
