# Supplementary figures and images for: miR-34a negatively regulates cell cycle factor Cdt2/DTL in HPV infected cervical cancer cells
Source: BMC Cancer. 2022 Jul 15;22:777. doi: 10.1186/s12885-022-09879-5 (PMC9288023; doi:10.1186/s12885-022-09879-5)

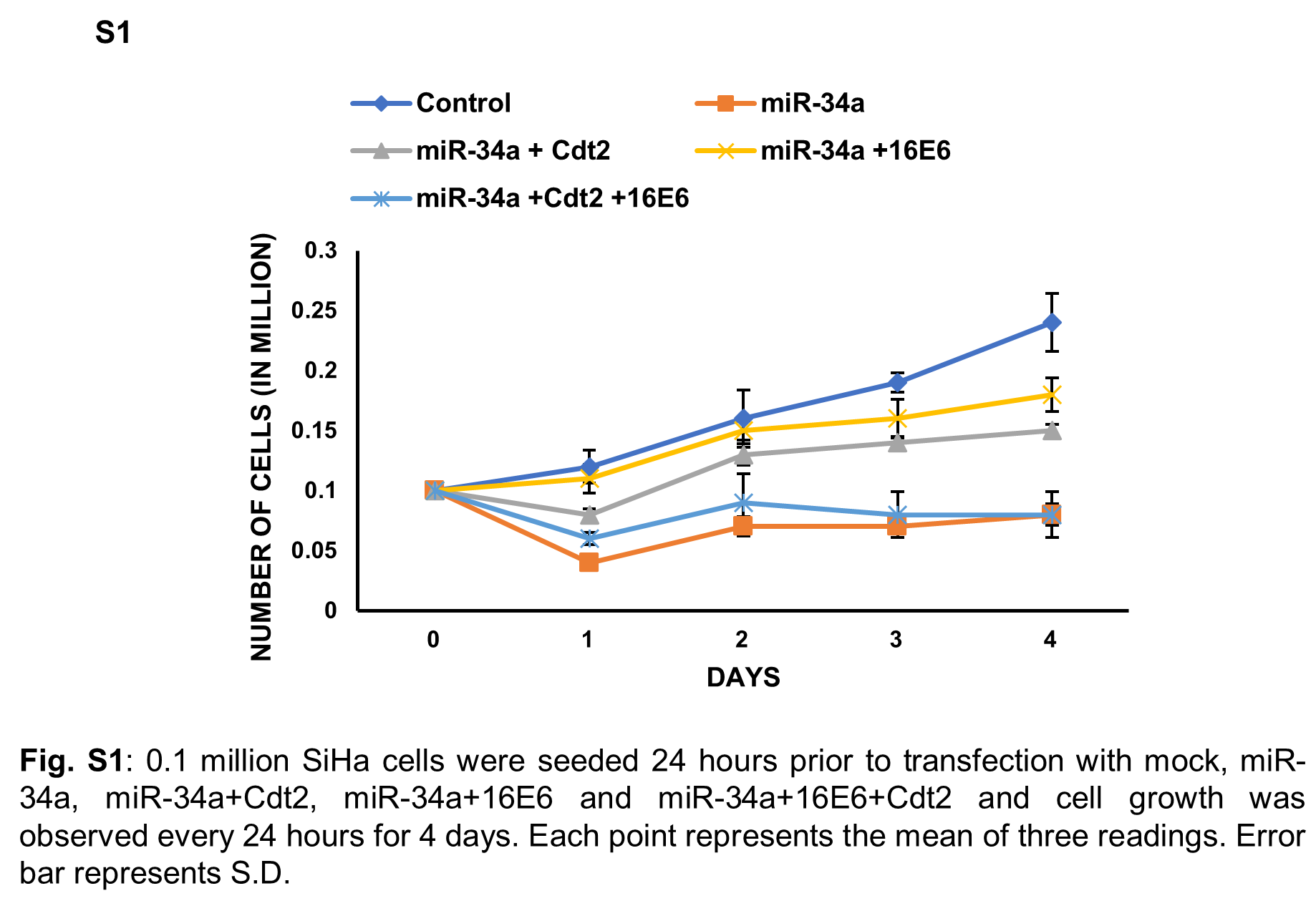

Supplement: Supplementary file 2 — Additional file 2. [file 12885_2022_9879_MOESM2_ESM.tif]

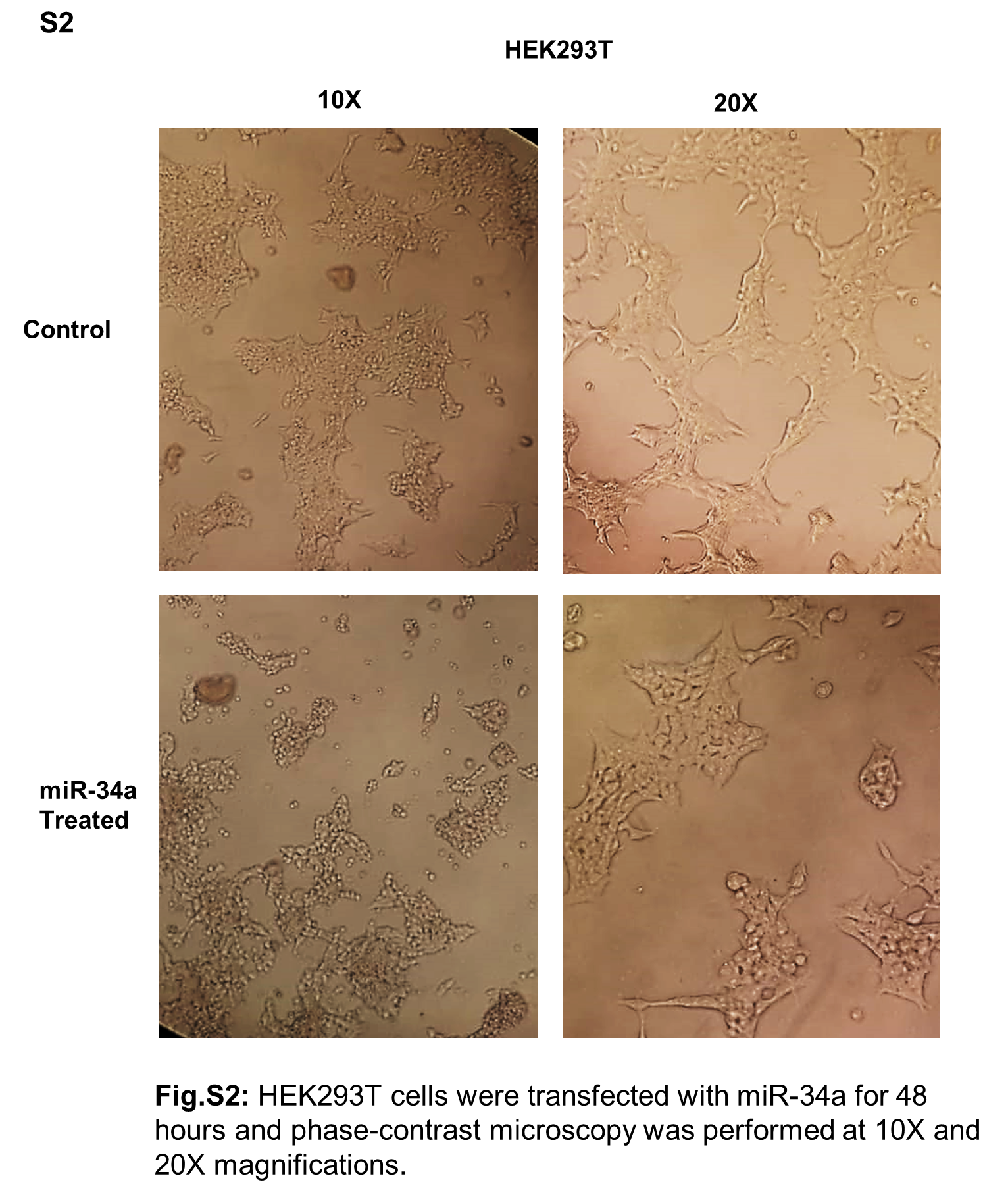

Supplement: Supplementary file 3 — Additional file 3. [file 12885_2022_9879_MOESM3_ESM.tif]
